# Toxicity evaluation of the contaminated area of Crotone from biological indicators: a multispecies approach

**DOI:** 10.1007/s10661-023-11056-5

**Published:** 2023-03-16

**Authors:** Anna Mastroberardino, Filomena Casaburi, Rosario Canino, Michelangelo Iannone, Salvatore Procopio

**Affiliations:** 1 Department of Physics, University of Calabria and National Institute for Nuclear Physics, Gruppo Collegato of Cosenza, Rende, Italy; 2 Regional Agency for Environmental Protection ARPACal, Catanzaro, Italy

**Keywords:** Contaminated sites, Phosphoric metasilicates, Ecotoxicological bioassays, Anthropogenic radioactivity, TENORM

## Abstract

Contamination of terrestrial and aquatic ecosystems by toxic industrial waste has become a major issue in many countries. Of particular concern is the reuse of toxic hazardous waste in construction materials. This paper examined for the first time the chemical and radiation ecotoxicity of site-specific Technological Enhanced Naturally Occurring Radioactive Materials (TENORM) residues from phosphate processing industry in soil environmental matrices through bioindicators. The area under investigation was the former industrial district of Crotone (Calabria, Italy), recently included within the Sites of National Interest (SIN), comprising the 42 Italian national priority contaminated sites. Major biological exposure pathways considered were absorption and bioaccumulation. The marine bacterium *Vibrio fischeri* and the freshwater crustacean *Daphnia magna* were employed as aquatic bioindicators, while for the soil ecosystem, the seeds of *Sorghum saccharatum* and *Lepidium sativum* were used. Selection of test species aimed at assessing the toxicity of wastes in soil as well as in freshwater or marine systems. Results indicated *V. fischeri* as the most sensitive of all the species tested (5.56 g/L), while *D. magna* was found to be affected at 94.27 g/L. An overall inhibition was observed in seedling growth as compared to control at the highest concentration of the pollutants (100 g/L), while seed germination was not adversely affected by the pollutant. At this preliminary level, data indicated a potential risk for biodiversity of the area. In fact, the measured toxicity thresholds, even if above 100 mg/L, are comparable to concentrations of the toxicants spread all over the territory of Crotone.

## Introduction

Big industrial centres near coastal areas have seriously damaged the quality of eco-systems, as is the case of the industrial plant district of Crotone, located on the east coast of Calabria, in Southern Italy. This province, one of the leading colonies of Magna Grecia in the Mediterranean Basin, is a recognized regional centre of biodiversity including numerous marine reserves and Special Protection Areas (Ministerial Decree, 1991).

In 1928, the City of Crotone became a major industrial center hosting two leading chemical plants, devoted to the production of zinc, phosphoric acid and complex fertilizer (Barone et al., 2010). In the beginning and for a long time, the enthusiasm for industrialization in a developing country prevented any concern for the environment and public health. Industrial activities ended in 1992, leaving the interested zone an industrial graveyard with huge amounts of waste left behind. During the last decades, phosphate residues have been partly used in this region as filling materials for the construction of roads and buildings, due to their mechanical properties, partly disposed in inert waste landfills, close to the seacoast, with unknown impacts to the environment and the health of the citizens (Barone et al., 2010; IAEA, 2003; Procopio & Nuccetelli, 2012; Andresz et al., 2019). Analysis carried out over the years on a wide collection of sediments of the Crotone industrial area evidenced heavy metal concentration values higher than the limits reported in D. Lgs. 152/2006 in soil and groundwater (Troisi et al., 2002) and in marine coastline (Cannata et al., 2016). Moreover, the exposure risk of polluted sediments to the fluvial and coastal dynamics possibly affecting the long-term survival of this ecosystem was evidenced in a recent study (Oliveri et al., 2022). The measured concentrations of certain contaminants in soil, compared to the Italian contamination regulatory limits for the commercial-industrial land use, are shown in Table 1.

**Table 1:** Soil highest concentrations of some heavy metals in the industrial area of Crotone

**Metal**	**Concentration (mg/kg)**	**Italian threshold value (mg/kg)**
As	2,682	50
Cd	2,173	15
Pb	37,700	1,000
Zn	80,020	1,500

In 1999, the national government declared a state of emergency in Calabria. This led to a widespread investigation on the illegal or improper disposal of wastes (Parliamentary Commission, 2011), and this region was included in the list of the *Remediation Sites of National Interest (SIN)*, due to the level of environmental contamination and health risk (Ministerial Decree no. 471/1999).

The term NORM (Naturally Occurring Radioactive Materials) refers to radioactive materials existing in the Earth’s crust normally. Technologically Enhanced Naturally Occurring Radioactive Materials (TENORM) are artificially concentrated NORM, where human activities have increased the potential for exposure compared with unaltered situations. In particular, TENORM wastes containing phosphorite rocks, as the ones contaminating the analysed Crotone area (Caridi et al., 2017), are characterized by the presence of radioactive phosphogypsum (PG) and metasilicates (Nero & Nazzarof, 1984; Stoulos et al., 2003), containing radionuclides from ^238^U and ^232^Th decay series which are of most radiotoxicity. Rare earth elements and barium are also enriched in the PG (Perez-Lopez et al., 2007). In Europe, the average concentration of natural uranium in soil is estimated to be approximately 2 parts per million, which is equivalent to 2 g of uranium in 1000 kg of soil (De Vos & Tarvainen, 2010), several orders of magnitude lower than uranium concentration of industry produced phosphate materials and related wastes (UNSCEAR, 2000).

Although the radiotoxic effects of uranium are often of considerable concern, its chemical toxicity effects may be dominant, also depending on the chemical compound involved and the enrichment grade. However, the variability of uranium concentration in sediments, due to its mobility and bioavailability in terrestrial environments, represents a source of several potential dangers. An example is represented by plant contamination, for which root transfer is the prevailing pathway (Paquet et al., 2009).

The hazardous wastes analysed in this study are a mixture of heavy metals and radioactive elements, managed onsite in buildings and spread around the study area at unknown concentrations. Previous studies have not attempted to quantify the risks to biota caused by the chemical and radiation releases in this region.

**Fig. 1:**
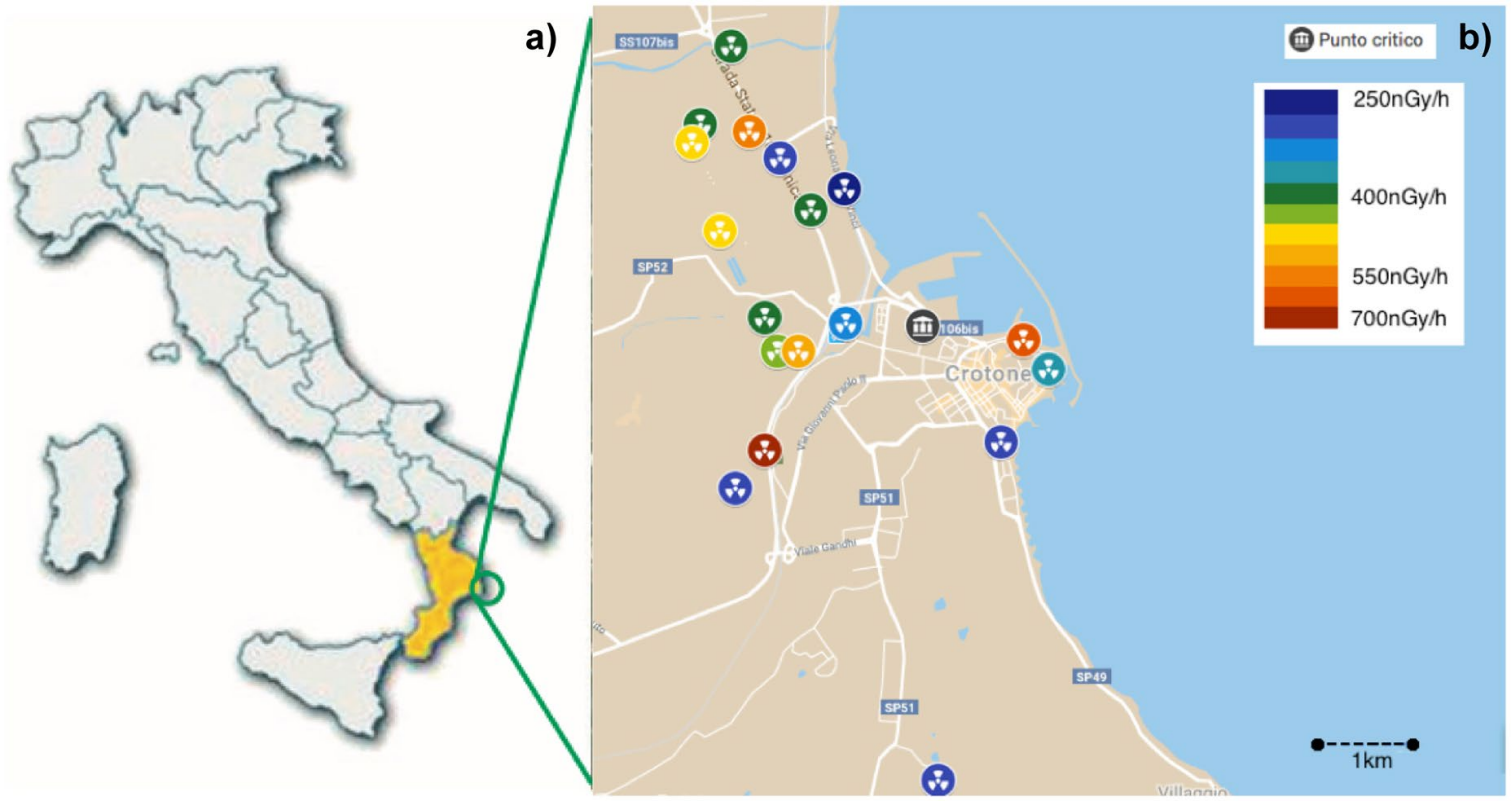
**a** The location of the region around the City of Crotone and **b** the map of contaminated sites, showing significant differences in dose rates within the affected area (Procopio et al., 2019a)

In 2019, the Regional Agency for Environmental Protection in Calabria (ARPACal), supported by the Prefecture of Crotone, published the first mapping results of TENORM occurrence within the area (Procopio et al., 2019a), in order to identify so-called hot spots. This term is used to describe points or areas where the radiation readings are significantly above normal background. The map resulting from the survey is shown in Fig. 1b and indicates the points where the presence of phosphorite waste was registered. Figure 1a illustrates the location of the site under study, where sediments were collected. It represents approximately 3% of the municipal area of Crotone (179,0.83 km^2^, ISTAT, 2017).

In this measurement (Procopio et al., 2019a), 16 hot spots were identified within the sampling zone. These included sites where the activity was > 250 nGy/h. The analysed residues showed high activity concentration of ^226^Ra (770–1200 Bq/kg) (Procopio & Nuccetelli, 2012), in secular equilibrium with its parent radionuclide ^238^U, and low activity concentrations of ^232^Th and ^40^K (UNSCEAR, 2000). After background radiation subtraction, a gamma dose rate of 434 $\pm$ 37 nGyh^−1^ at a height of one meter was measured in the study area, to be compared with an average background of 95 $\pm$ 15 nGyh^−1^ (Procopio et al., 2019a).

It is worth considering such data in relation to the observed uranium concentrations in the major phosphate rock deposits in East Africa (130–7800 Bq/kg) (Mwalongo et al., 2022). Similarly, high uranium concentration levels are measured in the US phosphate ores (260–3700 Bq/kg), with a radium concentration content in the phosphogypsum waste product varying approximately from 400 to 1300 Bq/kg (US EPA, 2008).

**Fig. 2:**
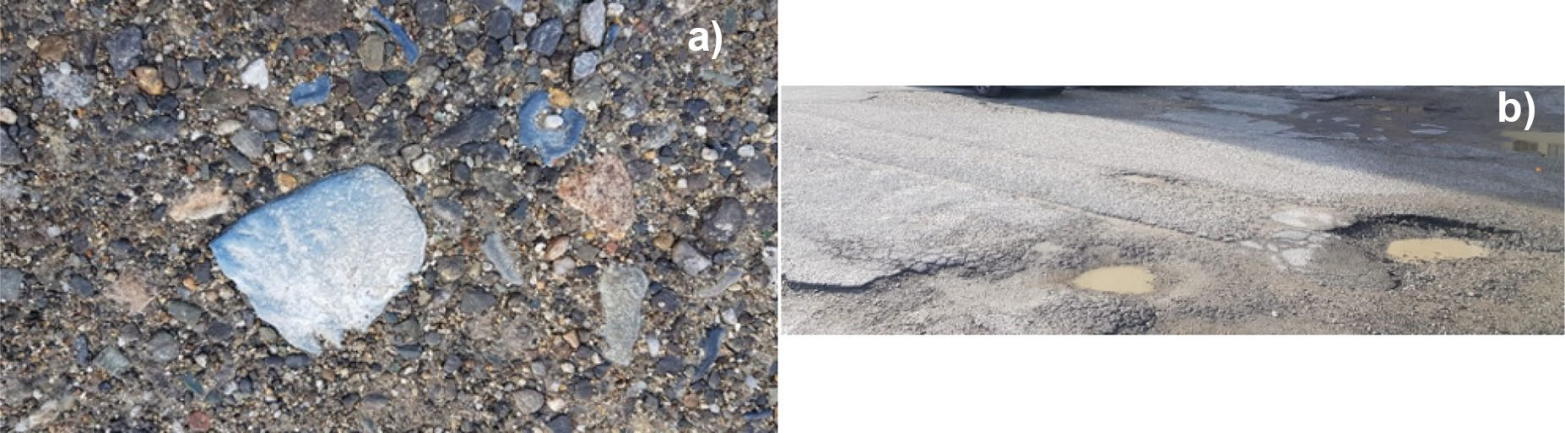
**a** Close-up picture of a road surface in Crotone showing a typical blue phosphate rock. **b** Severe potholes and slippage cracks in a major Crotone Road

Several epidemiological analyses (Frega et al., 2000; Zona et al., 2019) developed in recent years hypothesize a correlation between the increase in the incidence of respiratory pathologies and tumors of the respiratory tract, registered in this region, and toxic residues from the dismissed industrial installation. Regardless of the new remediation operations started in 2017, mainly concentrated in a minor portion of the contaminated site, the presence of road deposited hazardous sediments all over the urban areas (Fig. 2a) is particularly worrisome. Such a use of the territory, along with the detrimental status of asphalt pavements incorporating toxic wastes (Fig. 2b), poses a serious health risk to local residents and habitat.

The aim of this paper is to determine toxicity threshold levels for aquatic and terrestrial flora and fauna in the Crotone district. The assessment of hazardous levels through biological methods allows to take into account the whole content of pollutants, quantifying both radio and chemotoxicity and their synergetic interactions (Pandard et al., 2006).

In this analysis, three different bioindicators were considered: *V. fischeri* (Backhaus et al., 2000; ISO 11348, 2007) a marine bacterium whose bioluminescence inhibition is used in acute toxicity testing as a contamination endpoint; *D. magna* Straus (Sorvari & Sillanpaa, 1996; ISO 6341, 2012), a freshwater micro crustacean often used for acute immobilization toxicity testing in aquatic ecotoxicology; and seed germination rate and root elongation inhibition (Ratsch & Johndro, 1984; UNICHIM 1651, 2003), common bioassays in subchronic (72 h) phytotoxicity tests of water quality. For this test, the seeds of two species, *S. saccharatum* and *L. sativum*, were used to estimate the change in the phytotoxicity of amended samples in solid matrices.

To the best of our knowledge, this is the first ecotoxicological approach-based study for evaluating TENORM contamination in soil and water within the industrial district of Crotone, as an additional tool of search to the classical chemicals and physical approaches.

## Materials and methods

### Radiological and chemical waste characterization

Concerning the radiometric properties of the study area, a comprehensive literature review exists on the topic (Caridi et al., 2017, 2018; Procopio et al., 2019b) that provides accurate information about the presence and distribution of target contaminants in relation to background environment. The approach undertaken in these studies mostly makes use of a combination of ICP-mass and gamma ray spectrometry techniques to quantify the various radioelement concentrations in the zone. In later times, phosphorite TENORM waste from Crotone has been also characterized by means of X-ray fluorescence and X-ray diffraction analysis (Nicolino et al., 2022), in compliance with current regulations for assessing the associated radiation risk and planning the environmental restoration. The material element and mineral content obtained revealed a dominant quantity of iron (2570 $\mu g/g)$, significant amount of uranium ^238^U (7.4 $\mu g/g$) and of barium (362 $\mu g/g$), and a non-negligible occurrence of phosphorus (126 $\mu g/g$), despite the relative clean-up treatment performed on the harvested residues. The originating chemical reactions used to develop the phosphorite residues were also traced back (Nicolino et al., 2022).

A more systematic perspective was recently introduced enabling the determination of a radiometric survey map (Procopio et al., 2019a), in an unprecedented detail of the nature and overall extent of the contamination. The experimental setup used for the measurement included a dose rate monitor with beta/gamma probe for the first screening of the polluted sites. After the identification of a hot spot, high-resolution detectors such as sodium iodide NaI(Tl) spectrometers and surface contamination monitors were used to get details regarding radionuclides and activity levels. Real-time measurements for all detector channels were recorded and combined with a GPS for data compilation in order to map the resulting data stream of the screened region.

The radiometric mapping of the study area pursued the present ecotoxicological investigation to better quantify the health impact of the varying-toxicity blends spread all over this contaminated region, according to their concentrations.

### Soil sampling and preparation

The substances examined for their toxic properties were phosphorite (phosphoric metasilicates) residues from the district of Crotone. For the toxicological evaluation, sediment samples were collected at the different hot points in the sites undergoing the environmental remediation processes (Procopio et al., 2019a). Samples were taken from the soil and consisted of a solid granular medium with uniform density $\rho$ = 1220 kg/m^3^ and average grain size $\le$ 200 $\mathrm\mu$m.

### Design criteria for sampling

Two batches of specimen from the contaminated site were considered, with different composition:

- Unblended test medium, consisting of selected pure compounds of the phosphate residues.
- Blended test medium, with waste residues mixed with a known amount of inert material (sand), giving rise to a mixed low-level radioactive sample (silica sand acting as a shield for beta and gamma radiation).

The incorporation of inert material into the hazardous wastes was used to provide evidence of the relative chemical with respect to the radiation hazards and reproduce exposures to more realistic scenarios, with toxicants mixed to other materials.

From the two batches, three test samples were considered for the present study:

- A pure sample, consisting of homogeneous phosphorite residue with concentration equal to 10 g/L
- A weakly inert sample ( +), consisting of a compound of pure phosphorite and silica sand in the same percent composition (10 g/L)
- A heavily inert sample (+ +), made by pure phosphorite (10 g/L) in addition to a relevant amount of silica sand (50 g/L)

The sand samples were collected in a different location along the ionic coast, from the spots of a bathing beach showing the lowest values of natural radioactivity and heavy metal contents. Values of the sand profile analysis along all the coast are far below the recommended levels by the Italian legislation (Caridi et al., 2021). This allowed to exclude or consider as negligible the radiological and toxicity impact from the radiation shielding material to the sample results.

## Ecotoxicity tests

In this paper, the following tests were carried out, in accordance with standard guidelines. Prior to the study, initial range finding analysis were conducted. The concentration range of contaminant spanning from values completely ineffective to the selected endpoint up to lethal levels was considered and its limits used to define the toxicity interval. Preliminary analysis was also performed to determine whether any toxicity was being contributed by the procedural blanks. No toxicity was detected in these blanks.

Properties of the selected test protocols are described in Table 2.

**Table 2:** Properties of the selected bioassay tests used in this analysis

**Assay**	**Trophic level**	**Group of organisms/plants**	**Endpoint**	**Method**
V. fischeri*	Decomposer	Bacteria	Bioluminescence (EC_50_)	EN ISO 11348–3 2009
D. magna*	Primary consumer	Crustaceans	% Immobilization (24/48 h)	EN ISO 6341:2013
Phytotoxicity test**	Producer	Garden cress	Germination and root elongation (72 h)	UNICHIM 1651:2003

*Aquatic test

**terrestrial test

### *Vibrio fischeri* bioassay

In the first toxicity investigation, the commercial test system Microtox^®^ (Model 500 Analyser, AZUR Environmental) (Microbics, 1992), based on the 11348 EN ISO method (ISO 11348, 2007), was used to assess the inhibitory effect of the light emission of *V. fischeri*. The analysis was carried out in an accredited testing laboratory complying with ISO/IEC 17025.

The sample under test was incubated, in contact with the bacteria, in test tubes held in a water bath at 15 ± 1 °C, for 5, 15 or 30 min, and the luminescence intensity after incubation was evaluated, through a luminometer, with respect to the luminescence of pure bacteria. Reduction in light output provided estimation of sediment toxicity. Results were measured in terms of EC_50_, the percent concentration of the contaminant causing a 50% reduction in bioluminescence of the test organism with respect to a (non-toxic) blank under controlled conditions.

In this study, serial dilutions of the sample were prepared and tested for bioluminescence inhibition. The commercially available freeze-dried *Vibrio* strains were rehydrated prior to testing in reactivation solution. Starting from the primary dilution, a series of concentrations of the sample was prepared in diluent/control solution (2% NaCl in double distilled water). In order to identify the optimal dilution range for the target, we considered a preliminary solution with 500 mg of the tested sample dispensed into 50 ml diluent and performed tests in a dilution series (1:10). Within each test, five controls and five dilutions of the tested substance were used in two parallel replicates.

The inhibition effect of the tested toxicant resulting from the preliminary bioassay with a concentration of 10 g/L was:

- 96.67% for 15-min incubation time
- 97.86% for 30-min incubation time

The test results indicated a toxicity interval between 1 and 10 g/L.

The relative toxicity of the samples was expressed as a percentage of luminescence inhibition compared to the controls. The statistical endpoint of the test is the concentration of sample which is estimated to cause 50% inhibition of light production by the bacteria.

### *Daphnia magna* bioassay

The *D. magna* bioassay was undertaken according to standard test procedures dealing with environmental protection (ISO 6341, 2012). The organism used for this test belonged to the *D. magna* Straus species. This millimeter-sized crustacean is known to show relevant response to heavy metal pollution (lead, cadmium, zinc, copper) (Fargasova, 1994; Janssen et al., 2003). The purpose of the present test was to determine changes in *Daphnia* locomotor responses in the presence of toxicants and to measure the immobilization rate against the control.

The sample was checked for immobilized daphnids at 24 and 48 h after the beginning of the test. The obtained results were expressed as percentage of dead/immobilized individuals and in terms of EC_50_, the concentration of toxicant inducing the mobility inhibition of 50% of the essay. Test activation started with hatching of ephippial eggs of *Daphnia*, taking approximately 3 days to produce neonates to be used for the 24- or 48-h check. Stock cultures were maintained at a constant temperature of 20 $\pm$ 2 °C throughout the analysis, with a photoperiod of 16 h light and 8 h dark. Water was used for control and dilution. Due to the small size of the young born daphnids, a strip of black paper was used to considerably enhances the contrast between the test organisms and the white background of the light table, thus facilitating the visual observation of the test organisms. At the end of the test period, the sample was homogenized through slow manual shaking, followed by the measure and record of the number of dead and immobilized neonates versus that of the actively swimming test organisms. The mean and the % effect were calculated.

### Bioassay sensitivity testing

For both the first two bioassays, *V. fischeri* and *D. magna*, the performance of a reference test was advised in order to validate the correct execution of the laboratory procedure and the sensitivity of the test organisms (Buikema et al., 1982). The reference toxicant used in this study as a positive control was potassium dichromate, whose toxicity is well-known (Diamantino et al., 2000).

For the luminescent bacteria analysis using potassium dichromate as reference compound, the control test is valid if the reference substance causes 20 to 80% inhibition after a 30 min contact times at a concentration < 6 mg/L (ISO 11348, 2007).

Standard response of *D. magna* to toxicant (positive control) was verified as EC_50_ after 24-h exposure. The optimal conditions for this procedure are an illumination of at least 6000 luces and a temperature range of 20–25 °C. Results were compared to the expected EC_50_ according to the literature.

Following the acceptability criteria reported by OECD guideline (OECD, 2004), tests are considered valid if:

- The mortality in negative controls does not exceed 10% after 24 h of exposure without feeding.
- The EC_50_ recorded value of tested organisms in the quality control test is within the range 0.6–2.1 mg/L.

### Seed germination and root elongation bioassay

The seed germination and root elongation technique has been proven to be an established method for ecotoxicity studies of environmental samples of varying origin and composition (Truhaut, 1977; Hoffman et al., 2003; Nyholm, 1990).

The two different endpoints, seed germination and root elongation, were quantified in this study through an integrative indicator, the germination index (GI), in order to evaluate the effect of phosphorite residues mixed with sand on plant physiological processes. The GI represents the relative (sample over control) seed germination rate times the relative root growth, according to the equation:

(UNI 11357, 2010; Zucconi et al., 1985)

$$GI\%=\frac{GI_{sample\;\ast\;L_{sample}}}{GI_{control\;\ast\;L_{control}}}\bullet100$$

where *GI*_sample_ and *L*_sample_ are seed germination and root elongation (mm) values for the sample and *GI*_control_ and *L*_control_ the corresponding values for the control.

Results were based on two model species, namely *L. sativum* and *S. saccharatum*.

To evaluate the toxic effect of phosphorite on the seed germination process, seeds were sown on a paper substrate moistened with aqueous solutions at a concentration of 100 g/L. For the control, only distilled water was used. The tests were conducted using glass disposable Petri dishes (10 cm diameter) and a Whatmann n^o^1 filter paper disk. After determining the dry matter content of the three composts, the moisture content of the samples was standardized to saturation with double distilled water. After shaking, the flasks were centrifuged (and filtrated to minimize effects of suspended particles).

Four replicates of 10 undamaged seeds were placed uniformly on the surface of the filter paper, at the bottom of each disk, with 4 replicates for each pore water sample. The Petri dishes were sealed with parafilm to prevent evaporation and then placed in a dark controlled-environment growth chamber set at a constant temperature of 25.0 ± 0.5 °C without photoperiod. Seventy-two hours after the beginning of the incubation, percentage of germination was recorded and root length measured by means of a ruler to the closest millimeter. Seeds with a visible protrusion of the radicle, longer than 1 mm, were considered germinated (US EPA, 1996; Gong et al., 2001). In order for the test to be considered valid, seed germination rate beyond 90% was required for the reference substance. At the end of the test period, the number of germinated seeds was counted and the radicle and plumule lengths recorded. Sample GI values greater than control GI values indicate biostimulation, while sample GI values smaller than control groups suggest inhibition. Results of variance analysis allowed to check whether differences between the control and the different mixtures on germination percentage were statistically significant.

### Statistical analysis

For the Microtox test system, the calculations to estimate the EC_50_ and its 95% confidence limits were included in the Microtox Omni™ software provided by the manufacturer. For each test concentration, the Gamma function was calculated as (ASTM, 1995):

1 $$\Gamma_t=\frac{I_c}{I_t}\ - 1=\frac{H_t}{100-H_t}$$

with ${I}_{c}$ = the average light reading of filtrates of the control solution, ${I}_{t}$ = the light reading of a filtrate of a particular concentration of the test material and ${H}_{t}=$ the percentage inhibition.

From the results, a linear regression between the concentration (C) and the Gamma function was computed according to the following equation (ASTM, 1995):

2 $$log\Gamma=b\ast log\mathrm C+loga$$

In the above equation, *log a* is the intercept of the regression line with the ordinate at *log* $\Gamma$= 0, corresponding to $\Gamma$ = 1. Therefore, the EC_50_ can be derived from the antilog of the ratio of the intercept divided by the slope.

The measurements were carried out in four replicates. Linear regression analysis with the least-squares method was performed with Microsoft Excel. For each regression, the following information was provided: number of observations used in the analysis (N), coefficient of determination (${R}^{2}$), standard error of the estimate (S), Fisher’s criterion and significance. A probability value of *p* < 0.05 was considered statistically significant.

For the *D. magna* acute toxicity test, the log-normal model in the REGTOX software (Vindimian, 2005) for Microsoft Excel was used for the calculation of the dose–response parameters. The REGTOX software program is based on non-linear regression with Hill model to calculate the values. Effect concentrations (EC_50_) and their confidence intervals were estimated using the non-parametric bootstrap method.

For the seed germination and root elongation test, all experiments were performed in four replicates. Averages and standard deviations of the growth inhibition were calculated and fitted to the regression analysis. The averages of growth inhibition were compared by *T* test and *p* values were determined to evaluate the differences among treatments.

The germination test results are presented as average number of germinated seeds and standard deviation for each treatment.

## Results and discussion

**Fig. 3:**
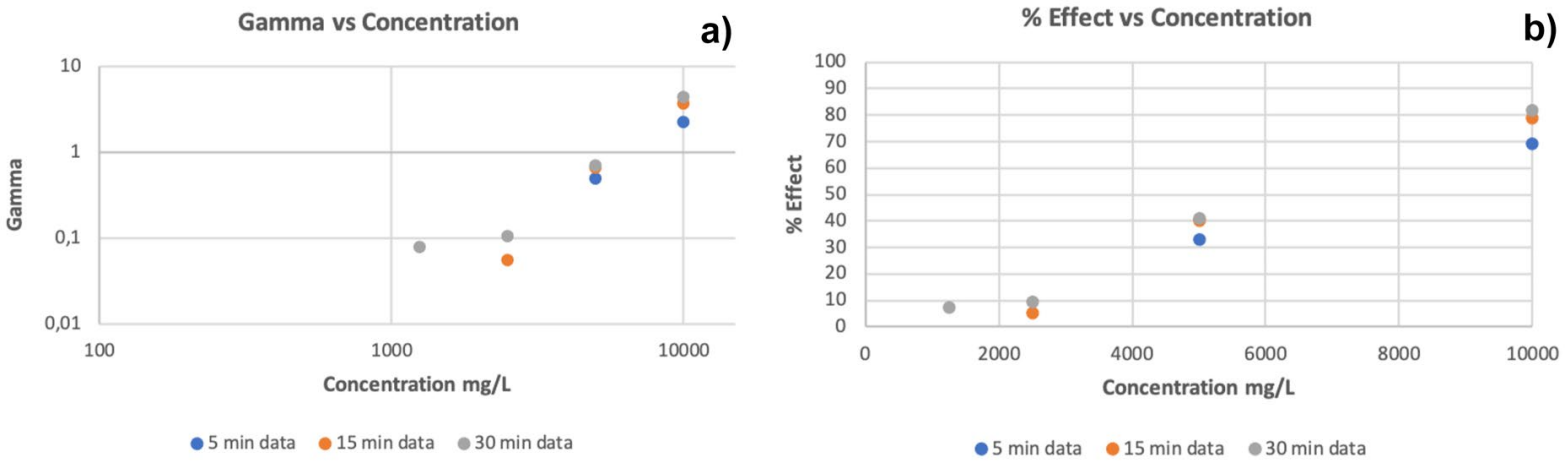
**a** Linear relationship between the function $\Gamma$ and concentration and **b** between the % effect and concentration according to Eq. 2 for the pure phosphorite test sample after all exposure times (5, 15 and 30 min)

For the *V. fischeri* test, linear regression distributions of observed $\Gamma$/concentration and % effect/concentration values were plotted to visualize the magnitude of toxicity change for a given compound. An example, as conducted herein, is given in Fig. 3 for values collected over the 5–15-30 min contact series for the pure toxic compound.

The resulting toxicity data within the chosen intervals of dilutions, in increasing order of toxicity and for incubation times of 15 and 30 min, are reported in Table 3.

**Table 3:** Relative toxicity of the samples expressed as a percentage of luminescence inhibition compared to the controls for the tests with the marine bacteria *V. fischeri*

Concentration g/L	**Test sample (pure)** Phosphorite 10 g/L		**Test sample ( +)** Phosphorite 10 g/L Sand 10 g/L		**Test sample (+ +)** Phosphorite 10 g/L Sand 50 g/L	
	**15 min** **% inhibition**	**30 min** **% inhibition**	**15 min** **% inhibition**	**30 min** **% inhibition**	**15 min** **% inhibition**	**30 min** **% inhibition**
0.625	−2.047	0.539	−6.726	−2.230	−3.351	2.542
1.250	0.184	7.270	−7.334	−1.933	−2.408	2.780
2.500	5.252	9.581	−1.384	5.421	−1.056	5.377
5.000	40.170	41.070	13.050	19.370	13.620	18.310
**10.000**	**78.740**	**81.630**	**54.860**	**57.540**	**51.220**	**55.450**

No significant differences were observed between 15 and 30 min exposure times, suggesting bacterial toxicity was complete after 15 min of exposure. The Microtox^®^ test outcomes indicated in all sample’s acute toxic effects on *V.fischeri* at the highest test concentration. The toxicity measured in the sample of pure phosphorite was significantly higher than that of the two samples of the mixed material and sand, which shared a similar degree of toxicity.

**Fig. 4:**
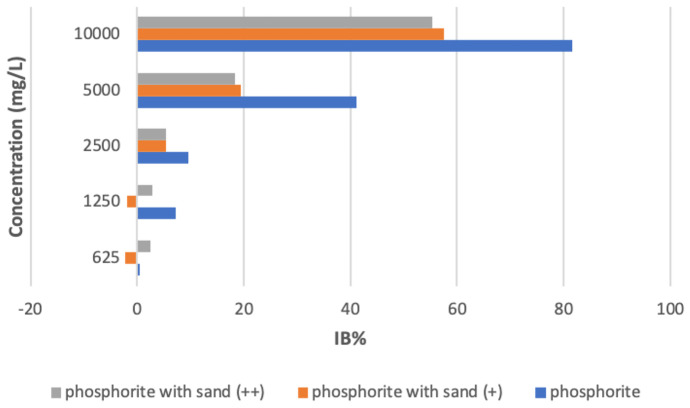
*V. fischeri* bioluminescence inhibition (I_B_%) after 30-min incubation time in various phosphorite residues

Figure 4 shows the bioluminescence inhibition of *V. fischeri* by the tested samples at 30-min exposure time. Significant differences of the responses of the strains to the addition of sand to the toxic sample extracts are visible from the comparison among the different compounds. In particular, 82% bioluminescence inhibition after 30 min exposure was recorded at the highest pollutant concentration. In other work (Mekki & Sayadi, 2017) reporting results on the usage of the luminescent bacteria for toxicity estimation of wastewater from the phosphate processing industry in a Mediterranean soil, a similar effect was measured.

The resultant EC_50_s of the analysed samples are displayed in Table 4.

**Table 4:** Acute toxicity data (95% confidence interval) of target compounds on *V. fischeri*

	**Compound**		
**EC**_**50**_ **(g/L)**	**Test sample (pure)** Phosphorite 10 g/L	**Test sample ( +)** Phosphorite 10 g/L Sand 10 g/L	**Test sample (+ +)** Phosphorite 10 g/L Sand 50 g/L
15 min EC_50_	6.22 g/L	9.38 g/L	9.82 g/L
30 min EC_50_ [95% CL]	**5.56 g/L** [1.23 – 25.12]	8.94 g/L [2.50 – 32.05]	9.31 g/L [2.03 – 42.54]

Since 15-min exposure data did not permit the development of concentration–response relationship (EC_50_ calculated from two data points), the associated values for the 95% confidence intervals were given only for 30-min exposure time data.

The 15 min EC_50_ values ranged from 6.22 to 9.82 g/L and the 30 min EC_50_ values from 5.56 to 9.31 g/L.

The present findings documented the toxic nature of all the analysed samples, showing that potentially harmful effects are reduced by the presence of sand addition. It is also worth emphasizing that the adverse effects of all compounds to luminescent bacteria is significant, as evidenced by about 51% inhibition of luminescence at 15-min exposure to the less toxic mixture.

The above results indicate toxicity for concentrations above 100 mg/L, thus representing in principle a low contamination risk for biodiversity with respect to several known pollutants. Anyway, concentration levels in the waste material hosted in the study area are comparable to and even greater than the measured thresholds.

The above data are in accordance with other work (Kim et al., 2013) using fish for the analysis of acute toxicity test of phosphate compounds, taking into account the fish and *V. fischeri* interspecies correlations in the toxicity analysis of numerous chemicals (Wang et al., 2016).

For a preliminary comparison between bioindicators, equal exposure concentrations of toxicants were considered during pre-tests. The results indicated that *D. magna* was much less sensitive to toxic effects than *V. fischeri* in all five reference toxicant concentrations. Henceforth, a test sample strongly enriched in phosphorite, up to a concentration of 100.000 mg/L, was deemed to be used for the *D. magna* bioassay. The compound samples were tested for toxicity at the following concentrations: 6.25%, 12.5%, 25%, 50% and 100%. For the five effect doses, the parameter values with their confidence intervals were given with two different significance levels, according to the user’s choice. The resulting % of immobilization for the 24- and 48-h measurements (Table 5) provided evidence of a dose–response effect in the highest-concentration sample. The obtained EC_50_ at 48 h for immobilization was 94.27 g/L.

**Table 5:** Mean immobilization (% of total organisms) of *D. magna* for exposure to toxicant concentrations up to 100 g/L

	*D. Magna* Mean Immobilization	
**Toxicant concentration (mg/L)**	**24-h exposure**	**48 -h exposure**
6250	0	0
12.500	5%	10%
25.000	5%	10%
50.000	10%	15%
100.000	35%	55%

This result is well above 100 mg/L and is consistent with previous work (Kim et al., 2013), where no *D. magna* immobility was observed up to 100 mg/L in the toxicity assessment of phosphate compounds.

The 24-h reference test with potassium dichromate carried out with *D. magna* provided an EC_50_ value of [0.65; 1.25]mg/L 95% CL. The observed percentage of immobilization in the negative control was 5%. Both recorded values validated the procedure.

The positive control with potassium dichromate run alongside *V. fischeri* tests gave EC_50_ = 3.4 mg/L with a percentage inhibition effect of 48%.

In this research, consistent results were achieved for each test control in accordance with the criteria for validity of the respective test guideline. A summary of the endpoint values of the different test models is reported in Table 6.

**Table 6:** Results of the reference tests performed on potassium dichromate with the ecotoxicological essays used in this analysis

**Organism group**	**Organism**	**Endpoint**	**Reference substance, validity range**	**Mean values** **(and SDs)**
Water flea	*D. magna*	EC_50_	Potassium dichromate, 0.6–2.1 mg/L	0.95 ± 0.30 mg/L
Luminescent bacteria	*V. fischeri*	% Immobilization (&& concentration)	Potassium dichromate, 20–80% < 6 mg/L	48% 3.4 $\pm 0.9$ mg/L

In the phytotoxicity tests, the effects of phosphorite residues on the seedling germination and growth were observed at the highest examined concentration of 100 g/L. The response of the toxicity of the compound is summarized in Table 7. Based on these values, the toxicity test outcomes showed similar trend but different magnitude of plant responses.

**Table 7:** Effect of toxicant at a concentration of 100 g/L on germination and early growth in *S. saccharatum* and *L. sativum* compared to the control. Results with SDs were from four replicated dishes, each containing ten seeds. Double-distilled water was used as control medium

**Bioassay response**	***Sorghum saccharatum***		***Lepidium sativum***	
	Control	Sample	Control	Sample
Average number of germinated seeds	9.3 $\pm 0.5$	9.3 $\pm 0.5$	9.9 $\pm 0.1$	9.8 $\pm$ 0.8
Root length (mm)	85.9 $\pm 11.7$	40.0 $\pm 13.3$	77.1 $\pm 3.4$	55.5 $\pm 0.3$ *
GI%	46.5%		71.3%	

Results showed that the compound significantly inhibited root elongation (*p* < 0.05, marked with *) of the *L. sativum* sample. Moreover, the compost toxicity appeared to have a major influence on the root length response variable.

According to Zucconi et al. (1985), GI lower than 50% indicate high phytotoxicity, values between 50 and 80% indicate moderate phytotoxicity, and values above 80% mean no phytotoxic material. In line with this, phosphorite at the tested concentration can be considered as highly phytotoxic for the *S. saccharatum* seed and moderate phytotoxic for the *L. sativum* tested seed.

**Fig. 5:**
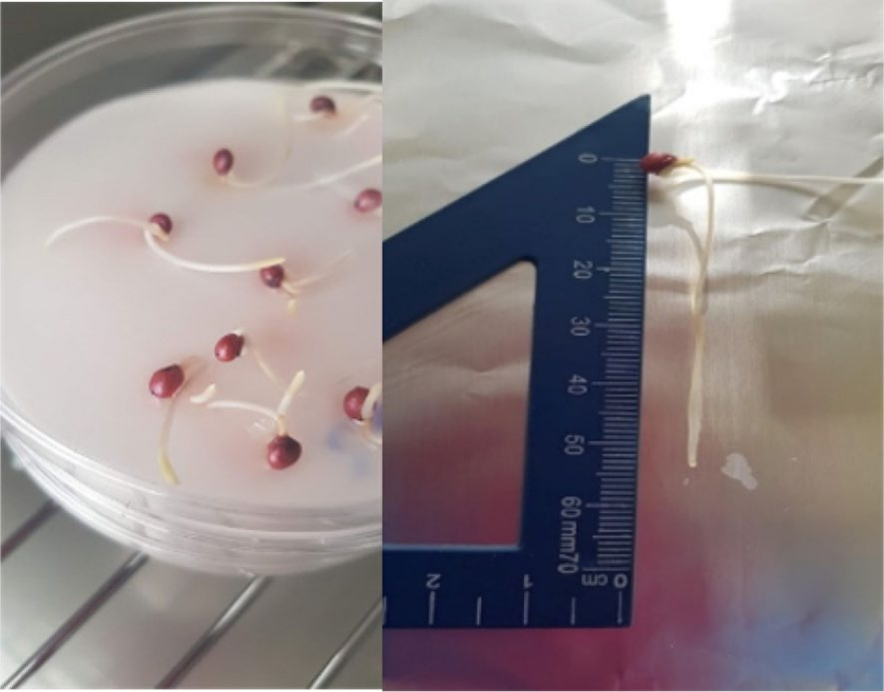
*Sorghum* seeds in Petri dishes

**Fig. 6:**
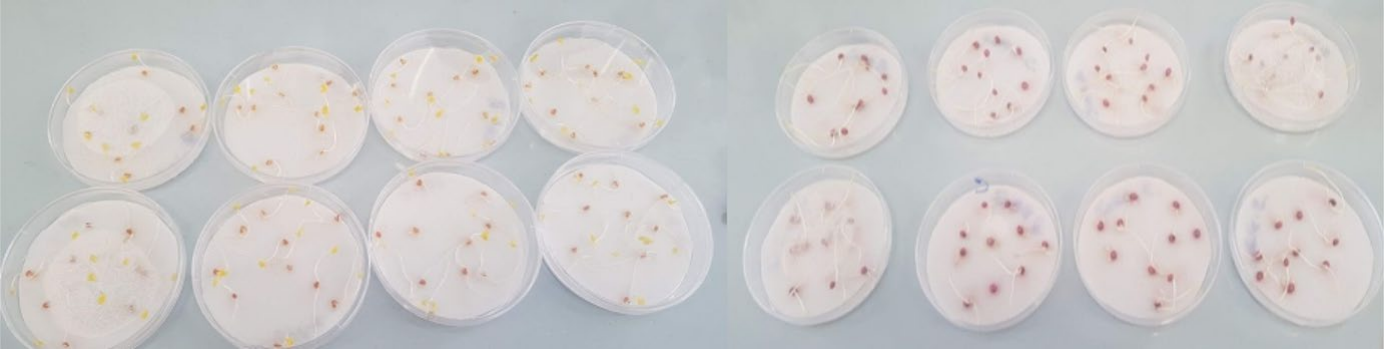
*L. sativum* in Petri dishes

**Fig. 7:**
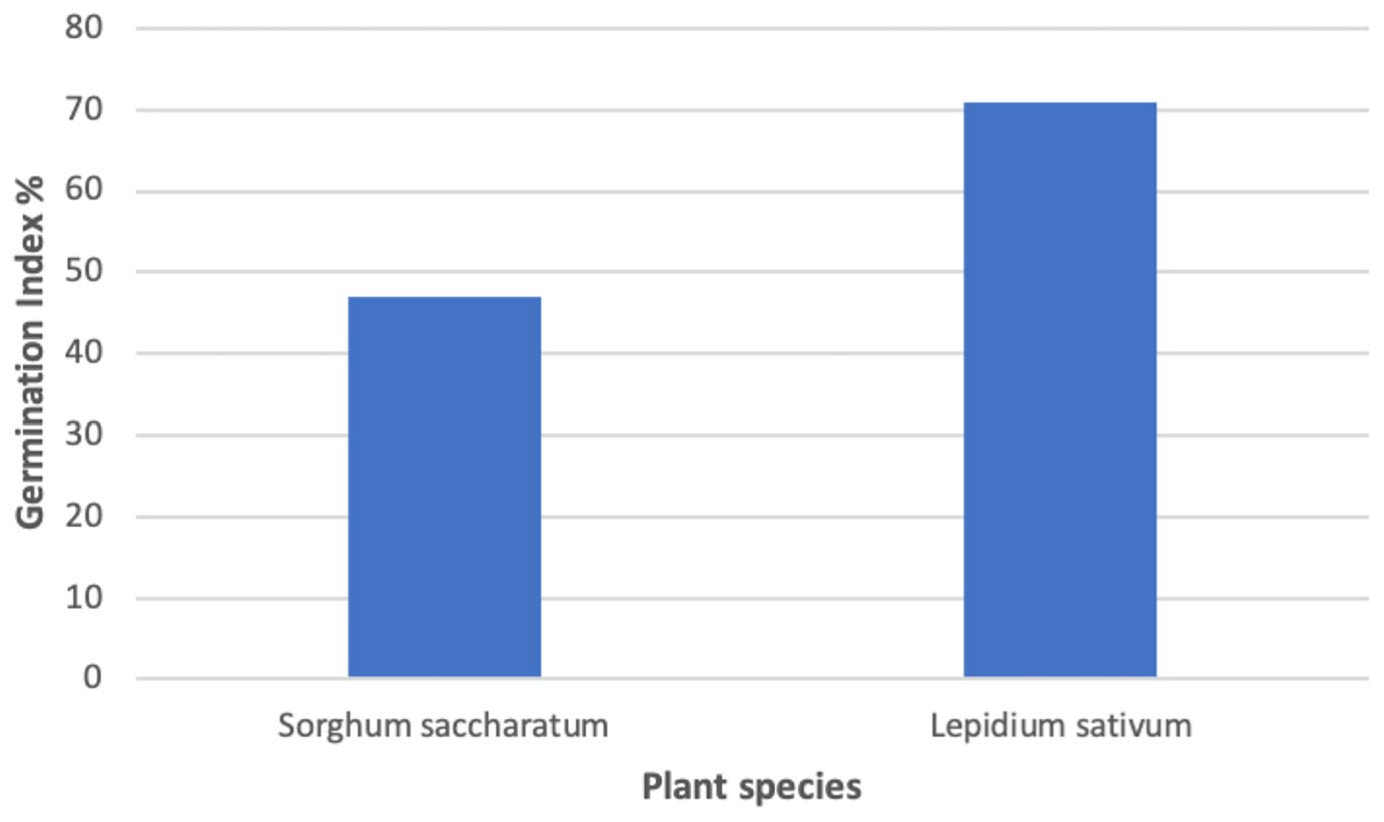
Mean percent germination over control of *Sorghum* and *Lepidium* seeds exposed to a 100 g/L phosphorite sample

The visual rating of the analysed compound toxicity on the tested plants is available in Fig. 5 for the *Sorghum* and in Fig. 6 for the *Lepidium* plant, respectively. Figure 7 illustrates the germination percentage of *Lepidium* and *Sorghum* seeds, respectively, exposed to the tested toxic sample of phosphorite with a concentration of 100 g/L. 

As general conclusions, the sediment did not prove to be toxic for the plant species germination up to a concentration of 100 g/L. At this level of content, the sample altered the root elongation of both the plant species to various degrees, inducing a statistically significant biosuppression in *L. sativum* growth. The germination indices of both *S. saccharatum* and *L. sativum* were inhibited by the sediment material.

Anyway, much experimental concerns on the results of phytotoxicity assays are mandatory, due to the rather high variability affecting analysis of data, such as (the level and rapidity of) seed germination. Therefore, these indications should preferably be confirmed testing more sediment samples, with a larger range of concentrations of these chemicals.

It has to be emphasized that in all of the ecotoxicological tests compared here, results refer to the mixture of contaminants rather than to individual components in untreated raw samples.

The interactive ecotoxicity of pollutant mixtures, such as hydrocarbons and heavy metals, have been widely studied. For example, Lors et al. (2010) investigated the degree of pollution of soil samples taken from different areas of an industrial site contaminated with polycyclic aromatic hydrocarbons (PAHs) and heavy metals. The test was carried out by means of test battery, including inhibition of lettuce germination and growth. In the soil sample mainly contaminated by organics and with low concentrations of metals, the germination of *L. sativa* was inhibited by 71% and its growth by 80%, while remaining low (13–20%) for the less PAHs contaminated soils. In this study, results of toxicity estimation were clearly correlated with the concentration of one of the contaminants in the presence of mixed pollution.

Ecotoxicity of wastes containing both chemicals and radionuclides has been evaluated more seldom. In such cases, results of toxicity estimation are not always clearly correlated with concentrations of chemicals or levels of radioactivity. For instance, when considering hazards associated with uranium, all its isotopes being radioactive, it is not possible to study chemical outcomes fully independently from radiation effects. Additionally, the non-threshold, stochastic effects of natural radiation make a quantitative comparison of chemo- and radiotoxicity difficult.

In Park et al. (2016), acute toxicity tests with *D. magna* were conducted on PG in soil mixed samples, labeled according to the waste concentration (i.e. PG50 indicates 50% PG with the remainder of soil). Concentrations of pollutants, including radioactive isotopes, were significantly lower than those affecting the district of Crotone. During a 48-h test, immobilization rates for PG30 and PG50 were 25% and 45%, respectively, and after 24 h, no mobilization of *D. magna* occurred with PG100. These data denote a more severe inhibitory effect than what recorded in the present work.

In this regard, the different related chemical properties associated with solubility of phosphate rocks and PG need to be considered. As evidenced in Kybartiene et al. (2015), phosphate minerals form aggregate crystals with very marginal solubility in water and in sulfuric and phosphoric acids, whereas PG is a fine-grained material with silty-sandy structure and highly solubility in water.

Phosphorite and its waste also exhibit different radiological properties, whose impact on this study needs to be further investigated. In the phosphate rock, the natural ^238^U and ^232^Th decay-series are in equilibrium with their progeny. During the industrial process, this equilibrium is disrupted, with radionuclides migrating to the final product. Wide variations in the average activity concentrations of ^238^U and ^226^Ra are thus detectable in phosphate rocks and its product sample (El-Bahi et al., 2017).

## Conclusions

This work is a first contribution to the evaluation of the chemical and radiation hazards of phosphate factories residues through different biological indicators. The investigation was carried out in the Crotone territory, a unique habitat where sediments, spread around the area at unknown concentrations, were also employed as inert material and partly brought to surface layers of urban roads.

Main objectives were to identify the toxicity threshold effect levels of contamination for the site-specific biota through the exposure routes of aqueous and soil pathways. For the scope, elutriate and contact bioassays were used.

Four different compounds were analysed, two with increasing inert material content, to possibly evidence chemical toxicity with respect to radioactive effects, and two with pure tested substances at very different concentrations. Results of the investigation confirmed that different organisms have non-identical sensitivity and response on the substances they are exposed to.

*V. fischeri* proved to be the more sensitive test, with a 30 min EC_50_ ranging from 5.56 to 9.31 g/L in the different compounds under study.

*D. magna* and phytotoxicity assays showed similar sensitivity to the toxic components of the analysed wastes. For the *D. magna* bioassay, the obtained EC_50_ at 48 h for immobilization was 94.27 g/L. At the same concentration value of 100 g/L, a germination index of 47% for *S. saccharatum* and of 71% for *L. sativum* was observed. Root elongation for both plant species was also affected by the toxic action of phosphorite, even if improved statistics is needed for more reliable results. The measured inhibition of bioluminescence in sediment elutriates with expected decreasing toxicity ranged from 81 to 51%, revealing the different potency of harmfulness through the addition of inert material to the pure pollutant, even if data were not clearly correlated with concentration levels of radioactivity in the waste samples studied.

Results are synthetized in Table 8. No previous studies have been performed with the same substances.

**Table 8:** Summary of the ecotoxicological endpoints used in this analysis to assess the hazardous properties of tested substances with their respective critical effect concentrations and duration of exposure

**Method**	**Assay**	**Endpoint** (exposure time)	**Result** (exposure concentration)
UNI EN ISO 11348–3 2009	*V. fischeri*	Bioluminescence (30-min EC_50_)	**5.56 g/L** (Phosphorite concentration = 10 g/L)
UNI EN ISO 6341:2013	*D. magna*	% Immobilization (48 h) 48 h EC_50_	**55.% 94.27 g/L** (Phosphorite concentration = 100 g/L)
UNI 11357:2010	*S. saccharatum*	IG% (72 h)	**46.50%**
	*L. sativum*		**71.30% **(Phosphorite concentration = 100 g/L)

Overall, toxicity was observed at concentrations above 100 mg/L; therefore, phosphorite residues in the study area are not expected to significantly affect the surrounding biota. Anyway, the peculiarity of this territory, hosting relevant amount of pollutants not covered with soil or any other material and incorporated into asphalts for roads, in concentrations comparable to, or even greater than the threshold levels found in this study, suggest a potential hazard for the habitat of the zone and its tailings. Moreover, the fine road dust contamination, due to land corrosion and degradation, and the possible ground water alteration, due to the close proximity of the toxic substances to aquifer, are main issues requiring a continuous monitoring of the zone in the coming years.

An improved characterization of the exposure is mandatory for these measurements in the future to understand contaminant risk, once a guidance harmful threshold level has been estimated.

## Data Availability

The datasets generated and analysed during the current study are available from the corresponding author on reasonable request.
